# Gadoteric Acid and Gadolinium: Exploring Short- and Long-Term Effects on Healthy Animals

**DOI:** 10.3390/jox15020034

**Published:** 2025-02-21

**Authors:** Susana Coimbra, Susana Rocha, Sofia D. Viana, Rute Rebelo, Petronila Rocha-Pereira, Irina Lousa, Maria João Valente, Cristina Catarino, Luís Belo, Elsa Bronze-da-Rocha, Flávio Reis, Alice Santos-Silva

**Affiliations:** 1 UCIBIO i4HB, Faculdade de Farmácia, Universidade do Porto, 4050-313 Porto, Portugal; srocha@ff.up.pt (S.R.); rutesarabrandao@gmail.com (R.R.); petrorp@ubi.pt (P.R.-P.); irina.filipa@hotmail.com (I.L.); cristinacatarino@ff.up.pt (C.C.); luisbelo@ff.up.pt (L.B.); elsa.rocha@ff.up.pt (E.B.-d.-R.); 2 UCIBIO i4HB, Translational Toxicology Research Laboratory, University Institute of Health Sciences (1H-TOXRUN, IUCS-CESPU), Avenida Central de Gandra 1317, 4585-116 Gandra, Portugal; 3 Institute of Pharmacology & Experimental Therapeutics & Coimbra Institute for Clinical and Biomedical Research (iCBR), Faculty of Medicine, University of Coimbra, 3000-548 Coimbra, Portugal; sofia_viana@estesc.ipc.pt (S.D.V.); freis@fmed.uc.pt (F.R.); 4 Center for Innovative Biomedicine and Biotechnology (CIBB), University of Coimbra, 3000-548 Coimbra, Portugal; 5 ESTESC-Coimbra Health School, Polytechnic Institute of Coimbra, 3046-854 Coimbra, Portugal; 6 H&TRC-Health and Technology Research Center, Coimbra Health School, Polytechnic University of Coimbra, 3046-854 Coimbra, Portugal; 7 Health Science Research Centre, University of Beira Interior, 6201-506 Covilhã, Portugal; 8 National Food Institute, Technical University of Denmark, 2800 Kgs Lyngby, Denmark; mjopo@food.dtu.dk

**Keywords:** contrast agents, kidney injury, inflammation, iron metabolism, dyslipidemia, hepatic disturbances

## Abstract

Regarding the safety of gadolinium (Gd (III))-based contrast agents, we aimed to evaluate the short- and long-term effects following a single exposure to gadoteric acid (DOTA) or to free Gd (III) using animal models. Biomarkers of kidney injury, inflammation, iron metabolism, dyslipidemia, hepatic and hematologic disturbances and kidney histopathological and differential gene expression (DGE) analyses were evaluated. In the short-term study, compared to the controls, exposure to Gd (III) was associated with higher inflammation; changes in lipid, iron and hepatic metabolisms; hematological alterations; and kidney damage. Exposure to DOTA revealed changes in hematological, lipid and hepatic biomarkers. In the long-term study, compared to the controls, exposure to Gd (III) or to DOTA showed much fewer changes than the short-term exposure. Comparing the kidney gene expression of Gd (III) or DOTA exposure versus the control, we found clearly different DGE patterns and a lower number of differently expressed genes in the long-term study, for both compounds. Our data show that a single-dose exposure to these compounds induces several short-term changes which over time return to normal or are sustained, although with less severity, especially in the case of DOTA.

## 1. Introduction

Gadolinium-based contrast agents (GBCAs) have been used for over 3 decades to improve the quality of magnetic resonance imaging (MRI). Since free gadolinium [Gd (III)] is toxic, exerting adverse effects on several organs and disturbing several physiological processes, this metal is stabilized by chelation to reduce its toxicity in GBCAs. Since Gd (III) and many of the GBCAs undergo renal excretion, the kidneys are one of the targets of their toxicity. GBCAs may lead to kidney injuries that, ultimately, may trigger the development of renal disease, especially in patients with renal impairment [1]. Chronic kidney disease (CKD), by enhancing retention of dissociated Gd (III), favors its deposition in renal tissue that may lead to the worsening of kidney disease and, ultimately, to nephrogenic systemic fibrosis (NSF) [2]. In postmortem studies of patients who died from NSF, Gd (III) deposition was found in several tissues, showing very high levels in the kidney, heart and blood vessels [3]. Most of the reported cases of NSF were associated with linear GBCAs [4]; the association of NSF with macrocyclic GBCAs were mostly reported in patients with mild and advanced renal disease [5,6,7,8], raising concerns regarding their nephrotoxicity [2,5,6,7,8]. The long-term toxicity of Gd (III) has been attributed, at least partially, to the slow release of ionic Gd (III) from the deposits in several organs [9,10,11,12]. Accumulation of Gd (III) in the kidney, as well as in other organs, has also been reported in individuals without renal dysfunction, mostly in those submitted to repeated exposure to GBCAs [13]. The amount of Gd (III) released from GBCAs and the extent of its deposition in tissues is unknown.

The mechanisms of Gd (III) toxicity in target organs are poorly clarified; however, upregulation of inflammation and oxidative stress, as well as metabolic dysfunction, affecting lipid and iron regulation, are pointed out as potential mechanisms involved in Gd (III) and GBCA toxicity [14,15,16,17,18]. Previous studies by our group, using human proximal tubular cells (HK2) found that Gd (III) exposure led to disruption in the oxidative status; mitochondrial dysfunction; cell death by apoptosis, switching to necrosis at higher Gd (III) concentrations; and autophagic activation. Likewise, disturbances in lipid metabolism and the upregulation of genes related to lipogenesis and lipolysis and of modulators of signaling pathways involved in the development and progression of renal disease were also found [19].

GBCAs are classified as linear or macrocyclic, depending on whether they have an open or an enclosing structure, respectively. Since Gd (III) dissociates more quickly and easily from linear chelates, they are considered less stable. Although considered more stable, macrocyclic GBCAs have also been associated with Gd (III) release and tissue deposition [20,21], namely in the liver and kidney, even after exposure to a single dose of a macrocyclic GBCAs [1].

The use in clinical practice of GBCAs in MRI studies, often repeated in a lifetime, deserves a more comprehensive approach in order to highlight the associated renal risks, given that CKD is a health problem worldwide and often undiagnosed in early stages [22]. Thus, considering the existent gaps of knowledge regarding the actual safety of GBCAs, our aim was to evaluate the short- and long-term effects associated with a single exposure of the macrocyclic GBCA, gadoteric acid (DOTA), and of free Gd (III), using an animal model. With that purpose, we evaluated early and traditional biomarkers of kidney damage, inflammation, iron metabolism, dyslipidemia and hepatic disturbances; hematological studies and kidney histopathological and differential gene expression (DGE) analyses were also carried out.

## 2. Results

The three sets of animals were matched for weight, both in the short-term exposure (control: 290 ± 6 g; Gd: (III) 304 ± 6 g; and DOTA: 289 ± 7 g; *p* = 0.186) and in the long-term exposure studies (control 333 ± 7 g; Gd (III) 329 ± 3 g; and DOTA 321 ± 9 g; *p* = 0.521). Given that hematological and biochemical parameters are influenced by sex and age [23], we used male Wistar rats and control groups of the same age in order to compare data at the two independent time-points after the administration of the compounds.

Considering the hematological evaluation (Table 1) 2 days after exposure to Gd (III), as compared to the control group, we found significantly higher numbers of neutrophils, and significantly lower numbers of basophils and platelets (PLTs), which also showed a significantly lower mean platelet volume (MPV); we did not find significant changes in the number red blood cells (RBCs). The group exposed to DOTA presented significantly higher RBC counts and a significantly lower erythropoietin concentration compared to the control group; moreover, compared to the Gd (III) group, the DOTA group showed a significantly higher number of PLTs associated with a significantly lower MPV and significantly lower numbers of neutrophils. Thus, excepting the RBC count and the erythropoietin concentration, the hematological values in the control and DOTA groups were similar.

Twenty weeks after exposure to the compounds (Table 1), the Gd (III) group, compared to the control group, presented a lower RBC count and hematocrit, a higher mean corpuscular hemoglobin concentration (MCHC) and a lower number of PLTs. For the animals exposed to DOTA, we only found a significantly lower PLT count as compared to the control group.

Considering the clinical biochemistry parameters, 2 days (Table 2) after Gd (III) exposure, animals presented higher levels of aspartate aminotransferase (AST), alanine aminotransferase (ALT), total cholesterol, low-density lipoprotein cholesterol (LDLc), ferritin and interleukin (IL6), and lower iron, urea and triglyceride (TG) levels, compared to the control group. For the DOTA group, as compared to the control, higher AST and lower TG levels were found. The levels of LDLc, iron, ferritin and IL6, which were significantly altered in Gd (III) group, were similar to those shown by the control.

In the long-term study (Table 2), the Gd (III) group, as compared to the control, presented significantly higher levels of AST, ALT, creatinine and iron, and significantly lower ferritin and cystatin C. The DOTA group showed significantly higher iron levels than the controls.

Considering the values found for the two groups of controls and the reference ranges presented by Patel et al. [23] for some hematological and biochemical parameters at the age of 8 weeks and more than 6 months old, most of the values found fall inside these intervals. For the other parameters evaluated in this study (and not considered by Patel et al. [23]), as far as we know, no reference values for Wistar rats at these ages were reported. Nonetheless, the interpretation of data in relation to reference values must be carried out with some caution. The statistical methodology associated with the establishment of reference values means that approximately 5% of healthy individuals will fall outside the reference values. Additionally, biological variation implies that some factors, such as genetics, diet, age, physical activity and animal breeders, among others, may cause variation in the values of the analytical parameters between individuals [24,25,26,27,28]. Besides differences in cohorts, methodology bias can greatly influence reference values and prevent them from being transferable between some laboratories. Indeed, some guides suggest that laboratories should establish their own reference values [29,30]. Moreover, our study population was about 1/10 of that in the work by Patel et al. [23], which may also account for the discrepancies.

The kidney weight did not differ between the sets of animals in both the short- (control: 1.57 ± 0.06 g; Gd (III): 1.59 ± 0.05 g; DOTA: 1.55 ± 0.07 g; *p* = 0.910) and long-term exposure studies (control: 1.66 ± 0.05 g; Gd (III): 1.79 ± 0.03 g; DOTA: 1.79 ± 0.06 g; *p* = 0.119).

**Figure 1:**
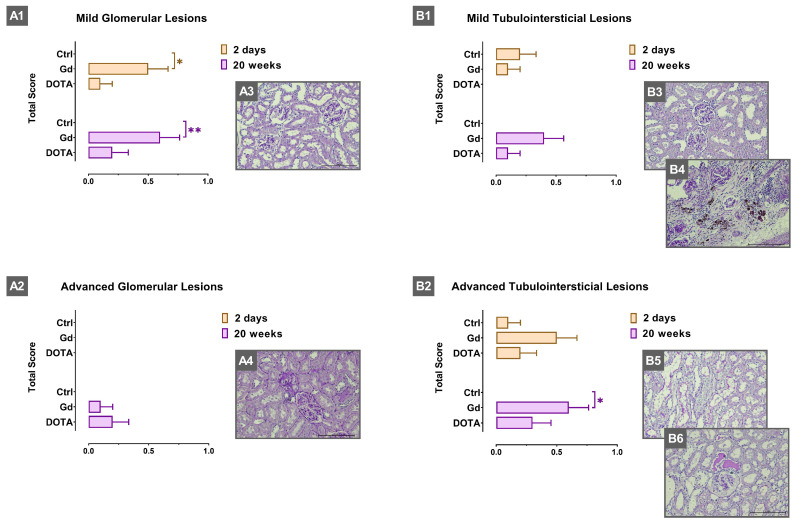
Total score of glomerular and tubulointerstitial lesions and representative images of some renal lesions observed in animals under study (PAS staining, 400×). The scale bar indicated in each image represents 100 µm. (**A1**) Total score for mild glomerular lesions observed in short-term (2 days) and in long-term (20 weeks) studies; (**A2**) total score for advanced glomerular lesions observed in short-term and in long-term studies; (**A3**) dilatation of Bowman’s space; (**A4**) glomerulosclerosis; (**B1**) total score for mild tubulointerstitial lesions observed in short-term and in long-term studies; (**B2**) total score for advanced tubulointerstitial lesions observed after 20 days or 20 weeks of exposure to compounds; (**B3**) interstitial inflammatory infiltrate; (**B4**) tubular dilation; (**B5**) vacuolar tubular degeneration; (**B6**) hyaline cylinders. Ctrl, control; Gd, gadolinium; DOTA, gadoteric acid. Results are presented as mean ± standard error of mean (SEM); * *p* < 0.05; ** *p* < 0.01.

Considering the histopathological analysis of the kidneys (Figure 1) in the short-term exposure study, the Gd (III) group presented a significantly higher total score for mild glomerular lesions than the control, and no advanced glomerular lesions were observed in the three groups under study. Moreover, no significant differences in the total score of mild and advanced tubulointerstitial lesions were observed in the Gd (III) group as compared to the control.

In the long-term study, the Gd (III) group showed significantly higher scoresof mild glomerular lesions and advanced tubulointerstitial lesions; the DOTA group did not present significant changes in the scores for glomerular and tubulointerstitial lesions (Figure 1).

**Figure 2:**
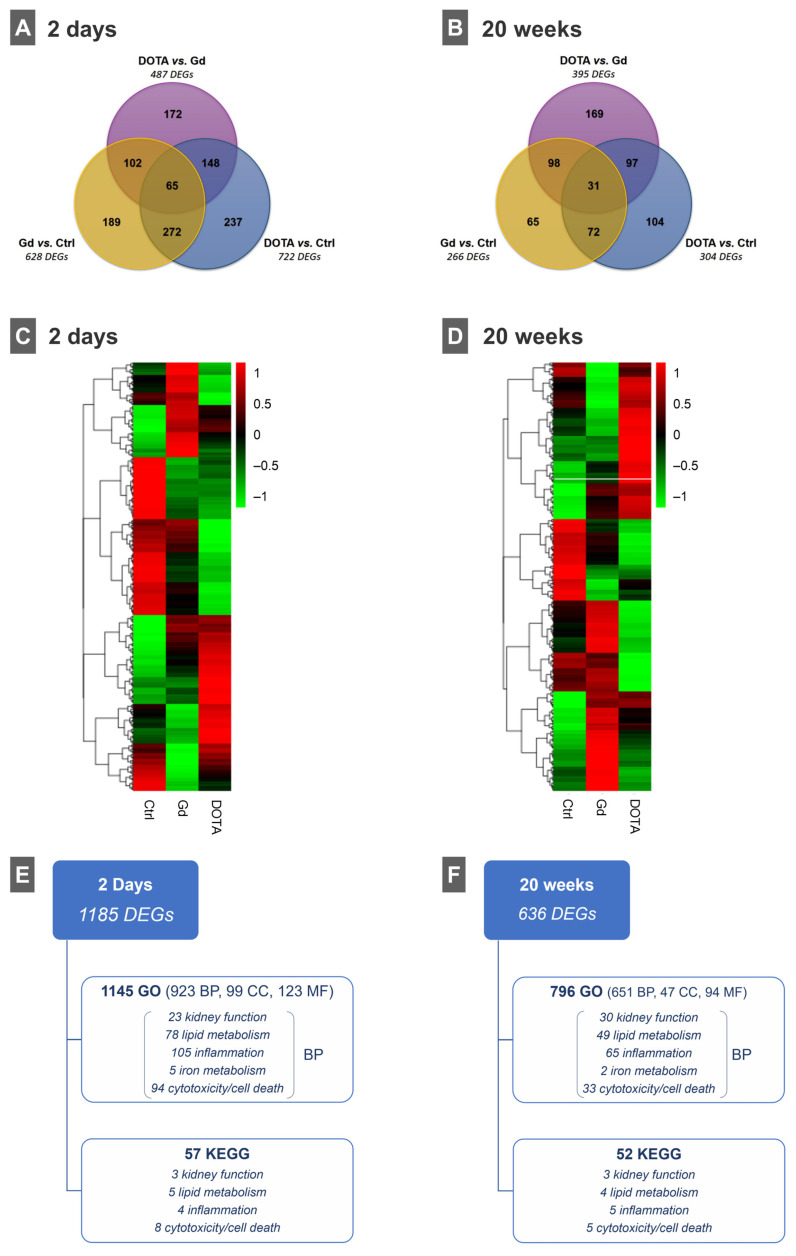
Venn diagrams of differentially expressed genes (DEGs) identified when comparing gene expression levels of Gd (III) vs. Ctrl, DOTA vs. Ctrl and DOTA vs. Gd (III) in (**C**) short-term and (**D**) long-term exposure studies. Heatmaps of gene expression levels of all identified DEGs in control (Ctrl), gadolinium (Gd (III)) and gadoteric acid (DOTA) groups (plots of hierarchical clustering of normalized FPKM values; red, high expression levels; green, low expression levels) for (**A**) short-term (2 days) and (**B**) long-term (20 weeks) studies. Functional/pathway enrichment analysis of all DEG results for (**E**) short-term and (**F**) long-term exposure studies (only terms with adjusted *p*-value < 0.05 are shown). BP, biological process; CC, cellular component; GO, gene ontology; KEGG, Kyoto Encyclopedia of Genes and Genomes; MF, molecular function.

When comparing the kidney tissue gene expression of the Gd (III) group versus the control, the DOTA group versus the control and the DOTA group versus the Gd (III) group, we found a total of 1185 and 636 differentially expressed genes (DEGs) in the short- and long-term exposures, respectively (with a Benjamini–Hochberg false discovery rate (FDR) < 0.05 and |log2 fold change| = 0; Tables S1 and S2). The Venn diagrams representing the number of unique and common DEGs between the group comparisons are presented in Figure 2 [A, short-term (2 days) exposure; B, long-term (20 weeks) exposure].

The gene expression levels of all of the identified DEGs are presented as heatmaps [Figure 2C, short-term (2 days) exposure; Figure 2D, long-term (20 weeks) exposure]; for both short- and long-term exposure, it is clear that the gene expression patterns are different between the Gd (III), DOTA and control groups.

To disclose the biologically relevant functional and signaling pathways, the enrichment analysis of all DEGs showed that these genes were significantly (*p*adj < 0.05) associated with 923 and 651 gene ontology–biological process (GO-BP) terms and with 57 and 52 KEGG (Kyoto Encyclopedia of Genes and Genomes) terms for the short- and long-term exposures, respectively (Tables S3–S6). Several of these terms were directly related to kidney function, lipid metabolism, inflammation, iron metabolism and general cytotoxicity/cell death process/pathways [Figure 2E, short-term (2 days) exposure; Figure 2F, long-term (20 weeks) exposure].

## 3. Discussion

Recently, it was reported that exposure to Gd_2_O_3_ in mice increased the number of monocyte-derived macrophages and myeloid-derived dendritic cells (M-DCs) in the liver; caused infiltration of neutrophils, M-DCs and B cells in the spleen; and increased the cell population of hematopoietic stem cells, multipotent progenitors and lymphoid progenitors in the spleen and the bone marrow [31]. Also, in peripheral blood, a decrease in the number of monocytes was found 2 days after exposure, and a decrease in total leukocyte and lymphocyte counts was found 7 days after exposure to Gd_2_O_3_ [31]. In male Wistar rats repeatedly exposed to gadodiamide, a decrease in the number of circulating reticulocytes and an increase in monocyte counts was observed [32]. After 2 days, the exposure to free Gd (III) was associated with higher neutrophil and lower PLT counts and with an increased MPV. After 20 weeks, Gd (III) exposure was also associated with a lower PLT count, as well as with erythrocyte disturbances, as suggested by lower RBC and hematocrit values, and increased MCHC. In the case of DOTA, short-term exposure was associated with higher RBC counts and lower erythropoietin levels; these alterations were no longer observed after 20 weeks (Table 1). Thus, our data suggest that exposure to Gd (III) seems to induce alterations in hematopoiesis affecting peripheral blood cell counts. Although no significant alterations in the RBC count were observed 2 days after exposure to Gd (III), its effects on the erythroid and platelet populations were more pronounced in the long-term study. A single dose 2 days after exposure to DOTA seems to favor an increase in the RBC count, increasing the partial pressure of oxygen and, therefore, decreasing the production of erythropoietin. However, DOTA exposure seems to have little effect on the peripheral blood cell count and erythropoietin levels after 20 weeks.

Gd (III) is known to accumulate in several organs and tissues, which favors the infiltration and activation of immune cells, triggering an increase in the circulating levels of inflammatory cytokines [31,33,34,35]. According to our data (Table 2), exposure to a single dose of Gd (III) induced, in the short term, an inflammatory response, shown by the higher levels of IL6 and ferritin and higher neutrophil count, that was no longer observed in the long-term study. Regarding DOTA, no significant alterations were found in the evaluated inflammatory biomarkers. Inflammatory signals may lead to changes in the hematopoietic system, such as increased granulocytic cell proliferation; DNA damage, triggering different forms of cell death; and changes in the bone marrow microenvironment, which can cause disturbances in hematopoiesis [36]. Thus, the inflammation observed after Gd (III) exposure may explain the blood cell count data. Upregulation of inflammation has been pointed out as a mechanism of Gd (III) cytotoxicity [37], but a single exposure to Gd (III), in case of normal renal function, does not seem to lead to long-term inflammatory disturbances.

The liver is known to be one of the main target organs of Gd (III) toxicity [12,31,32]; in accordance, we found evidence of liver damage 2 days after exposure to Gd (III) and DOTA, as shown by the significant increase in AST and ALT (Table 2). However, at 20 weeks, only the exposure to Gd (III) was still associated with increased levels of those liver damage biomarkers; the exposure to DOTA no longer had any influence on transaminase levels.

In both in vitro and in vivo studies, exposure to Gd (III) has been associated with changes in lipid, amino acid and carbohydrate metabolisms, both at the renal and hepatic levels [16,19,38,39,40]. Actually, alterations in renal metabolic profiles were reported, regardless of unchanged rat kidney histological analysis; the modifications in the hepatic metabolism are those that are typically observed in cases of acute liver injury [38]. It seems reasonable to hypothesize that the alterations in the metabolic pathways observed in the affected organs may have repercussions on the circulating levels of the metabolic biomarkers. According to our data, in the short term, exposure to free Gd (III) induced prompt changes in the lipid profile (with a higher level of total cholesterol and LDLc, and a lower level of TGs), while exposure to DOTA was associated with few disturbances in lipids and lipoproteins (only lower TG levels) (Table 2).

It was reported that exposure to Gd (III) causes disturbances in the hepatic nitrogen metabolism, with a decrease in trimethylamine N-oxide as well as in glutamate and glutamine, which are known to be involved in the transport of ammonia and in transaminase reactions [38]. These disruptions may explain the lower levels found for urea, which were not accompanied by significant changes in the other studied renal biomarkers, 2 days after exposure to Gd (III) (Table 2). Alterations in the levels of lipid profile and urea were no longer observed in the long-term study, suggesting that the impact on lipid and urea metabolisms was attenuated.

According to our study, exposure to Gd (III) was associated with a higher total score of mild glomerular lesions (Figure 1(A1)), although without significant changes in renal function, as suggested by the levels of the glomerular filtration biomarkers, creatinine and cystatin C (Table 2). A study by Gao et al. reported that seven days after exposure to Gd_2_O_3_, its biodistribution in the mice’s kidneys was imperceptible [31]. Data indicate that a single dose of Gd (III), in animals with normal renal function, in the short-to-medium term, does not interfere with glomerular function. Indeed, studies on rats with mild CKD have shown renal histological injuries without significant changes in the renal function biomarkers, creatinine and cystatin C [41]. Concerning exposure to DOTA in the short term, it did not induce significant changes in the levels of the studied kidney function biomarkers, nor in the histopathological analysis scores (Table 2 and Figure 1), suggesting a better renal profile for DOTA.

At long-term exposure to Gd (III), lower cystatin C levels were found, associated with no alterations in urea and with higher creatinine levels. Data suggest that cystatin C levels may be altered by several stimuli, and that different roles and/or regulatory mechanisms of cystatin C might exist in different cell types and tissues [42]. For instance, cystatin C produced by hematopoietic cell lineages was found to contribute significantly to cystatin pools [42]; in addition, after exposure to Gd (III), we observed, in the long term, a reduction in the peripheral number of some blood cell lines (RBC and PLT counts) alongside lower cystatin C levels. Published data also suggest that cystatin C is regulated at both transcriptional and post-translational levels. For instance, a mechanism for its post-translational regulation proposes the digestion of this cysteine protease inhibitor by proteases of another family, namely metalloproteinase (MMP)-2 and cathepsin D [42]. Exposure to Gd (III) was associated with the upregulation of MMP-1 [43] and increased mRNA expression of MMP-13 and -9 [44]. It is also known that Gd (III) is capable of long-term selective accumulation in lysosomes in vivo, and its accumulation in the lysosomes of liver macrophages leads to their damage [34]. GdCl_3_ induced labilization of liver lysosomes, increasing free activity of cathepsins B and L [45]. Thus, the decrease in cystatin C may be a long-term effect of exposure to Gd (III), a consequence of post-translational regulation by other proteases released/activated as a result of tissue injury by Gd (III) deposition. Creatinine is a classic biomarker of the glomerular filtration rate, and the higher values observed in the long term, after exposure to Gd (III), may result from glomerular function compromise; accordingly, we observed that, after 20 weeks, exposure to Gd (III) was associated with a higher mild glomerular lesions total score (Figure 1(A1)). Creatinine levels are dependent on age, race, sex and muscle mass. As Gd (III) seems to be retained in skeletal muscles [46], it cannot be ruled out that Gd (III) may interfere with muscle metabolism, contributing to increased creatinine levels.

Considering that kidneys are the main route for the excretion of Gd (III) and of most GBCAs, the proximal tubules are also a target of toxicity, making the tubular cells more prone to the toxic effects of Gd (III) [37,47,48]. In accordance, we found that exposure to Gd (III) was associated with a higher total score value for advanced tubulointerstitial lesions than that of the control group (Figure 1(B1)). Data obtained from the kidney histopathological analysis and creatinine levels suggest that, in the long term, Gd (III) may cause kidney injury and interference with renal function, which can be more pronounced in the case of multiple exposures to this agent or in the case of pre-existing renal impairment. A better renal profile for DOTA was also observed at 20 weeks after exposure to a single dose of the GBCA (Table 2 and Figure 1).

Gd (III) and GBCA toxicity has also been associated with iron metabolism disruption. We found changes in iron status biomarkers (higher ferritin and lower iron levels) 2 days after exposure to Gd (III), as well as an increase in the inflammatory biomarkers, as already mentioned (Table 2). An increase in ferritin, an acute phase protein, has been described [18,49] as a consequence of the upregulation of inflammation that occurs after exposure to Gd (III). Inflammation, namely IL6, also stimulates the production of hepcidin, the major regulator of iron metabolism, capable of binding to ferroportin at the membrane surface of enterocytes, hepatocytes and macrophages, inducing its internalization and degradation and thus inhibiting iron intestinal absorption and reducing iron mobilization [50]. Hepatic upregulation of hepcidin and downregulation of ferroportin after exposure to Gd (IIII) has been reported [35]. In vitro studies have shown that cell exposure to Gd (III) favors iron uptake [49], which may also have an impact on iron levels in vivo.

At 20 weeks, a single exposure to free Gd (III) was still associated with changes in iron status biomarkers, since lower ferritin and higher iron levels were observed, despite the normal values of the inflammatory biomarker IL6. In a study conducted on an animal model of CKD (induced by nephrectomy), 21 days after surgery, animals were exposed to a single dose of DOTA, showing, 2 days later, a significant decrease in total iron binding capacity and a significant increase in transferrin saturation, as well as a trend towards increased iron levels, which indicates higher iron availability; an increase in ferritin values was also described [18]. In our study, healthy animals, with normal renal function, exposed to a single dose of DOTA, also presented an increase in iron levels (Table 2). It was reported that after exposure to GBCA, Gd (III)–ferritin nanoparticles are formed, with Gd (III) binding, even at low concentrations of Gd (III), to the surface region of the oxyhydroxide core of ferritin [51]. The complex of Gd (III) with the metalloprotein ferritin was pointed out as a potential contributor to Gd (III) tissue deposition [51]. The heavy/light chain ratio in ferritin differs according to the type of tissue; heavy chains are more frequent on ferritin present in tissues characterized by rapid iron uptake and release (as kidneys and brain), while light chains are associated with with long-term iron storage (as in liver and spleen). According to the authors, these differences may influence Gd (III) tissue deposition [51]. Considering these findings, the development of potential new contrast agents has been conceived; Gd (III) nanoparticles encapsulated within a human heavy chain of ferritin nanocage is an example [52]. It is possible to hypothesize that the formation of Gd–ferritin complexes could have an impact on the circulating levels of free ferritin and iron, leading to higher iron levels and lower ferritin levels. This interference may affect the availability of iron for erythropoiesis, since we observed a decrease in RBCs and hematocrit 20 weeks after exposure to Gd (III). Our data show that Gd (III) and GBCAs interfere with iron metabolism, although it cannot be ruled out that biological variation factors (such as age and genetics) may be impactful on the values found [53]; therefore, further studies are necessary to better understand the interplay between Gd (III), iron and iron-binding proteins.

Since kidney deposition of free Gd (III) released from GBCAs is associated with molecular mechanisms of (renal) toxicity, as extensively reported [1,2,9,20,37], we performed whole transcriptome sequencing of kidney tissue to investigate dysregulated genes (DGE), biological pathways and networks (GO/KEGG) involved in the short- and long-term exposure to these compounds. At both time-points, we found several DEGs and very different molecular responses to the Gd (III) and DOTA groups when compared with the control and with each other, as can be seen by the gene expression heatmap patterns (Figure 2C,D); moreover, the enrichment analysis of these DEGs identified several of these genes as significantly associated with biological processes/pathways (Figure 2E,F). Of these, a total of 305 (33.0%) and 179 (27.5%) GO-BP terms and 20 (35.1%) and 17 (32.7%) KEGG terms, at 2 days and 20 weeks, respectively, were directly associated with kidney metabolism and/or function, lipid metabolism, inflammation, iron metabolism and general cytotoxicity/cell death process/pathways. The total number of DEGs also differed between short-term (1185 genes) and long-term (636 genes) exposure, showing that fewer genes were differentially expressed at 20 weeks, suggesting changes in tissue recuperation and metabolic processes normalization. Employing whole transcriptome analysis to the study of GBCAs, although still scarce, can be a useful tool to identify novel toxicity biomarkers. In fact, we are only aware of one other work that performed transcriptomics to cover this issue. Richter et al. evaluated the effect of a single dose of the GBCA gadodiamide on mice’s cerebella and found no significant DEGs or treatment-related clustering 4 weeks after injection [54]; this could be due to the time-point of the sacrifice used, as in the present work, we observed that the number of DEGs considerably decreased over time. Our data show that a single-dose administration of free Gd (III) or DOTA induced distinct gene expression patterns and regulation of signaling pathways in the kidneys, according to the compound and the time after exposure. A more comprehensive transcriptomics study is currently under preparation for publication.

In summary, although a single exposure to DOTA appeared to be safer than Gd (III), the toxicity of DOTA in the long term deserves further study, especially in the case of repeated exposure to this agent. The few studies evaluating the safety of the use of DOTA in repeated exposures showed that DOTA led to renal leukocyte infiltration, renal tubule atrophy and increased caspase 3 expression [55]; triggered hepatocellular necrosis and apoptosis, by damaging hepatocytes [12]; induced hippocampal gliosis and increased oxidative stress and inflammation in the brain [15]; was less efficiently removed from central nervous system, skin and renal tissues than gadoteridol, another macrocyclic GBCA [56,57]; and, although associated with an increased leukocyte count and Gd (III) tissue deposition after 5 weeks of exposure, these effects were less pronounced than for linear GBCAs [48]. We must emphasize that the use of different lengths of exposure to DOTA and different protocols makes data interpretation and comparison between studies not straightforward.

Our study also presents some limitations, namely the low number of animals used, despite being in accordance with the results of a power analysis performed to establish the optimal animal group size. The pharmacokinetic and toxicokinetic effects of the studied compounds may differ between animals and humans, and, thus, data found for Wistar rats may not predict the effects of this compounds on humans. Moreover, better knowledge regarding the Gd (III) and GBCA mechanisms of toxicity, namely nephrotoxicity, may be achieved through in vitro studies, and this topic deserves future studies. Data reported suggest that in humansrepeated exposure to GBCAs in the case of renal insufficiency may potentiate Gd (III) effects. The effects of repeated exposure to GBCAs, particularly in cases of mild renal disease, often undiagnosed, deserve further studies.

## 4. Conclusions

Our data show that, in healthy rats, a single-dose exposure to free Gd (III) or DOTA, in the short term after administration, induced an inflammatory response; changes in lipid, iron and hepatic metabolisms; hematological changes; and kidney cell injuries, while DOTA led to fewer changes (hematological, lipid and hepatic). In the long-term study, considerably fewer changes were observed for both Gd (III) and DOTA exposure. Kidney tissue gene expression studies for Gd (III) and DOTA showed clearly different DGE patterns, and a lower number of DEGs in the short- and long-term studies, for both compounds. In summary, despite the significantly safer profile for DOTA, further studies are necessary to clarify the impact of this GBCA, specifically regarding repeated exposure and pre-existent renal function impairment.

## 5. Materials and Methods

### 5.1. Animal Experimental Protocol

Eight-week-old male Wistar rats (Charles River Laboratories, Barcelona, Spain) were enrolled in this experimental design. During the protocol, animals were maintained in ventilated cages, under controlled temperature (22 °C) and humidity (50–60%), subjected to 12 h dark/light cycles and given free access to rat laboratory chow (4RF21 Mucedola, Milan, Italy) and tap water. This project received approval (#7-2022, on 28 November 2022) from the local (iCBR-FMUC) Animal Welfare Body (ORBEA) and from the Portuguese Authority (DGAV). All animal procedures carried out in this study were performed in accordance with the Animal Care National (DL 113/2013) and European (2010/63/EU) Directives, and the report followed the ARRIVE guidelines [58].

The rats were randomly divided into six groups (*n* = 10 each). In both the short- and long-term exposure studies, 3 sets of animals were exposed to a single dose (0.1 mmol/kg) of Gd (III) (in the salt form of GdCl_3_·6H_2_O), DOTA or vehicle (saline solution; control group) by tail vein injection. The protocol lasted for 2 days or 20 weeks after administration of the compounds or vehicle, for evaluation of the short- or long-term effects, respectively.

### 5.2. Sample Collection

At the end of the protocol, animals were sacrificed using an overdose (80 mg/Kg) of intraperitoneal pentobarbital (Sigma-Adrich, Saint-Louis, MO, USA). Blood was collected from the left ventricle, by cardiac puncture, into tubes without and with anticoagulant (K_3_EDTA) to obtain serum, whole blood or plasma. After perfusion with saline solution, kidneys were removed, cleaned, weighed and processed for histopathological and DGE analysis. Aliquots of serum, plasma and kidney (in RNAlater^®^, Sigma-Aldrich, Merck KGaA, Darmstadt, Germany) were immediately stored at −80 °C until assayed.

### 5.3. Assays

Hemogram was assessed in whole blood (K_3_EDTA) using an automated blood cell counter (HORIBA ABX, Amadora, Portugal). The serum levels of creatinine, urea, AST, ALT, iron, TGs, total cholesterol, high-density lipoprotein cholesterol and LDLc were evaluated using routine automated methods (Siemens Healthineers, Erlangen, Germany). Serum ferritin was measured by immunoturbidimetry (Siemens Healthineers, Erlangen, Germany). The concentrations of erythropoietin, cystatin C, oxidized LDL (oxLDL) and IL6 were analyzed through rat specific enzyme-linked immunosorbent assays (ELISA), according to the manufacturer’s instructions (Rat Erythropoietin SimpleStep ELISA Kit, Abcam, Cambridge, UK; Rat cystatin C ELISA kit, Rat IL6 HS ELISA kit, Rat oxLDL, MyBiosource, San Diego, CA, USA).

For histopathological analysis of the kidney, samples were fixed in neutral formalin 10% and embedded in paraffin wax and 4 μm thick sections were stained via periodic acid–Schiff (PAS) method. All samples were examined via light microscopy using a Zeiss Axioplan 2 microscope (Carl Zeiss Microscopy, LLC, New York, NY, USA) and images were captured using a Leica DFC450 digital microscope camera (Leica Microsystems, Wetzlar, Germany). For evaluation of kidney lesion severity, glomerular and tubulointerstitial lesions were identified and evaluated on the full tissue on the slide. Briefly, glomerular and tubulointerstitial lesions were divided into mild and advanced groups. Mild glomerular damage was assessed by evaluating thickening of Bowman’s capsule, glomerular hypertrophy, hypercellularity and dilatation of Bowman’s space; advanced glomerular damage was assessed by evaluating glomerular atrophy, glomerulosclerosis and glomerular “blebbing”. Mild tubulointerstitial lesions included tubular dilation, tubular basement membrane irregularities, tubular atrophy and interstitial inflammatory infiltration; advanced tubulointerstitial lesions were assessed evaluating the presence of hyaline cylinders and vacuolar tubular degeneration [41]. The median score for each lesion was given by the average of the individual animal scores. Glomerular hypertrophy was assessed by measuring, in each rat, the areas of ten cortical and the areas of ten glomeruli of the cortico-medullary junction, randomly chosen. A semiquantitative rating was used: 0—absent/normal (<5%); 1—mild (5–25%); 2—moderate (25–50%); 3—severe (>50%). The final score of each lesion was obtained after averaging the individual scores of each animal [41].

For DGE analysis, phenol–chloroform total RNA extraction from kidney tissue was performed, according to manufacturer’s instructions (TRI Reagent^®^ Protocol, Sigma-Aldrich). Briefly, after thawing, 1.0 mL of TRI reagent solution was added to 100.0 mg of tissue and then homogenized, utilizing a Potter tube and blender for cell lysis. Chloroform was added and, after centrifugation (12,000× *g*, 15 min, 4 °C), RNA was precipitated with isopropanol, from the aqueous phase. Samples were again centrifuged (12,000× *g*, 10 min, 4 °C), and the RNA pellet was resuspended and washed in 75% ethanol (7500× *g*, 5 min, 4 °C). Finally, the RNA pellet was dissolved in RNase-free water and stored at −80 °C until further assayed. RNA concentrations were measured via NanoDrop (ND-1000 Spectrophotometer, NanoDrop Technologies Inc., Wilmington, DE, EUA) and RNA integrity/quality was assessed utilizing Invitrogen™ Qubit™ 4 Fluorometer with Invitrogen™ Qubit™ RNA IQ Assay (Invitrogen, Waltham, MA, USA) to assess the minimal requirements of purity and quality of the RNA samples for further transcriptome sequencing. The samples that did not meet the purity requirement were purified/concentrated with GRS Pure RNA kit (GRiSP, Lda., Porto, Portugal). Subsequent mRNA sequencing and data analysis was performed by Novogene Co., (Cambridge, UK), as a procured genomic service. The samples analyzed were triplicates of a total RNA pool from all samples of each study group (6.0 µg, OD 260/280 and 260/230 ≥ 2.0, IQ ≥ 7.0). RNA-seq library preparation and sequencing (reference genome: ncbi_rattus_norvegicus_gcf_015227675_2_mratbn7_2), preprocessing of sequencing data, gene expression quantification (FPKMs—Fragments Per Kilobase of transcript sequence per Millions base pairs sequenced), DEG identification [DESeq2 package; Wald test and *p*-values adjusted using the Benjamini-Hochberg FDR method; considering the criteria threshold for significantly DEGs of: FDR < 0.05 and |log2 fold change| = 0] and DEG clustering [mainstream hierarchical clustering of log2(FPKM) values and normalization of the rows (Z-score)] were executed according to standard methods by Novogene Co. Functional/pathway enrichment analysis (GO and KEGG pathways) was performed for each exposure time-point, respectively, utilizing the total DEGs encompassing the Gd (III) versus control, the DOTA versus control and the DOTA versus Gd (III) comparisons [only terms with adjusted *p* value (*p*adj) < 0.05 were considered significant]. The NovoMagic (Novogene^TM^) online tool was used for heatmap graphics and Venn diagram plotting.

### 5.4. Statistical Analysis

Statistical analysis was performed using the IBM Statistical Package for Social Sciences (SPSS) for Windows, version 29.0 (IBM, New York, NY, USA). Shapiro–Wilk analysis was used to determine if data displayed a normal distribution. Differences between groups were evaluated using Kruskal–Wallis test (followed by post hoc pairwise comparison or by Mann–Whitney U test two-by-two comparisons, whenever statistical significance was observed) or the one-way ANOVA test (supplemented with Bonferroni post hoc). Results are presented as mean ± standard error of the mean (SEM). Statistical significance was accepted at *p* < 0.05.

## Figures and Tables

**Table 1:** Hematological evaluation for animals exposed to gadolinium (Gd) or gadoteric acid (DOTA) and for control group (Ctrl) after short-term (2 days) and long-term (20 weeks) exposure to the compounds.

	2 Days			20 Weeks		
	Ctrl (*n* = 10)	Gd (*n* = 10)	DOTA (*n* = 10)	Ctrl (*n* = 10)	Gd (*n* = 10)	DOTA (*n* = 10)
RBC (×10^12^/L)	7.83 ± 0.11	7.99 ± 0.20	8.27 ± 0.09 aa	8.40 ± 0.14	7.87 ± 0.12 a	8.00 ± 0.07
Hb (g/L)	155.2 ± 1.4	159.7 ± 4.0	164.1 ± 1.8	160.0 ± 2.2	153.6 ± 2.3	154.3 ± 1.4
HCT (L/L)	0.44 ± 0.01	0.45 ± 0.01	0.46 ± 0.01	0.44 ± 0.01	0.41 ± 0.01 a	0.42 ± 0.01
MCV (fL)	55.67 ± 0.46	54.78 ± 0.58	55.54 ± 0.36	52.91 ± 0.42	52.35 ± 0.51	53.33 ± 0.73
MCH (pg)	19.83 ± 0.17	19.54 ± 0.24	19.85 ± 0.19	18.91 ± 0.09	19.52 ± 0.16	19.46 ± 0.24
MCHC (g/dL)	35.32 ± 0.15	35.69 ± 0.21	35.76 ± 0.23	36.06 ± 0.23	37.31 ± 0.26 a	36.53 ± 0.37
RDW (%)	18.06 ± 0.24	18.95 ± 0.40	18.49 ± 0.21	18.99 ± 0.30	19.37 ± 0.47	19.15 ± 0.55
WBC (×10^9^/L)	5.00 ± 0.19	4.99 ± 0.13	5.35 ± 0.40	4.55 ± 0.25	3.81 ± 0.22	4.56 ± 0.31
NEUT (×10^9^/L)	0.10 ± 0.01	0.24 ± 0.03 aaa	0.15 ± 0.01 bb	0.18 ± 0.01	0.15 ± 0.01	0.16 ± 0.01
EO (×10^9^/L)	0.013 ± 0.002	0.017 ± 0.002	0.014 ± 0.002	0.021 ± 0.002	0.017 ± 0.004	0.024 ± 0.003
BASO (×10^9^/L)	0.024 ± 0.003	0.018 ± 0.003 a	0.021 ± 0.002	0.027 ± 0.003	0.024 ± 0.002	0.023 ± 0.002
LYMPH (×10^9^/L)	4.78 ± 0.18	4.68 ± 0.12	5.11 ± 0.39	4.26 ± 0.23	3.58 ± 0.22	4.30 ± 0.30
MONO (×10^9^/L)	0.067 ± 0.002	0.060 ± 0.004	0.066 ± 0.008	0.053 ± 0.004	0.042 ± 0.004	0.047 ± 0.003
PLT (×10^9^/L)	467. 4 ± 12.8	304.0 ± 37.5 aaa	439.1 ± 12.5 bb	523.5 ± 20.8	426.5 ± 19.aa	424.9 ± 10.6 aa
MPV (fL)	6.22 ± 0.09	6.92 ± 0.16 aa	6.20 ± 0.05 bbb	6.51 ± 0.09	6.64 ± 0.10	6.37 ± 0.13
Epo (pg/mL)	18.13 ± 1.17	10.69 ± 2.84	8.25 ± 1.95 aa	9.90 ± 1.57	12.12 ± 2.32	13.01 ± 1.51

BASO, basophil; EO, eosinophil; Epo, erythropoietin; Hb, hemoglobin; HCT, hematocrit; LYMPH, lymphocyte; MCH, mean corpuscular hemoglobin; MCHC, mean corpuscular hemoglobin concentration; MCV, mean corpuscular volume; MONO, monocyte; MPV, mean platelet volume; NEUT, neutrophil; PLT, platelet; RBC, red blood cell; RDW, red cell distribution width; WBC, white blood cell. Results are presented as mean ± SEM. a *p* < 0.05 vs. Ctrl group; aa *p* < 0.01 vs. Ctrl group; aaa *p* ≤ 0.001 vs. Ctrl group; bb *p* < 0.01 vs. Gd group; bbb *p* ≤ 0.001 vs. Gd group for each time-point exposure.

**Table 2:** Biochemical evaluation for animals exposed to gadolinium (Gd) or gadoteric acid (DOTA) and for control group (Ctrl) after short-term (2 days) and long-term (20 weeks) exposure to the compounds.

	2 Days			20 Weeks		
	Ctrl (*n* = 10)	Gd (*n* = 10)	DOTA (*n* = 10)	Ctrl (*n* = 10)	Gd (*n* = 10)	DOTA (*n* = 10)
Urea (mg/dL)	35.10 ± 1.24	29.56 ± 1.09 a	32.00 ± 1.55	26.30 ± 1.75	26.10 ± 1.15	29.50 ± 1.33
Creatinine (mg/dL)	0.37 ± 0.01	0.35 ± 0.01	0.33 ± 0.02	0.16 ± 0.03	0.26 ± 0.03 a	0.15 ± 0.02 b
Cystatin C (μg/mL)	0.81 ± 0.06	0.98 ± 0.08	0.83 ± 0.08	1.05 ± 0.08	0.73 ± 0.05 a	0.83 ± 0.05
AST (IU/L)	60.60 ± 2.17	78.90 ± 3.32 aaa	74.00 ± 2.57 aa	39.22 ± 3.51	81.33 ± 8.49 aaa	43.33 ± 5.29 bb
ALT (IU/L)	24.88 ± 0.93	30.44 ± 1.43 a	28.50 ± 1.59	16.67 ± 2.48	54.00 ± 9.94 aa	17.89 ± 2.73 bb
TG (mg/dL)	213.1 ± 16.3	115.2 ± 9.4 aaa	129.6 ± 18.0 aa	123. 3 ± 17.28	95.9 ± 8.2	130.3 ± 12.0
Total Chol. (mg/dL)	50.60 ± 2.43	63.70 ± 3.67 aa	58.80 ± 1.50	37.30 ± 3.99	44.00 ± 2.45	43.40 ± 4.60
HDLc (mg/dL)	21.50 ± 1.38	22.80 ± 1.15	23.80 ± 0.96	10.20 ± 1.22	13.10 ± 0.46	12.70 ± 1.46
LDLc (mg/dL)	4.50 ± 0.31	8.20 ± 0.49 aaa	5.00 ± 0.30 bbb	4.10 ± 0.43	3.25 ± 0.25	3.70 ± 0.42
oxLDL (ng/mL)	27.28 ± 0.88	30.11 ± 2.73	31.06 ± 2.19	20.0 ± 0.8	20.39 ± 0.95	18.08 ± 0.84
Iron (μg/dL)	172. 4 ± 4.2	134.2 ± 7.1 aa	165.3 ± 8.1 bb	83.0 ± 8.0	136.5 ± 6.2 aa	118.5 ± 12.8
Ferritin (μg/L)	30.2 ± 4.0	138.9 ± 22.4 aaa	48.6 ± 4.5 bbb	249.7 ± 17.3	68.3 ± 6.6 aaa	180. 6 ± 38.7 b
IL6 (pg/mL)	1.77 ± 0.26	3.03 ± 0.32 a	1.68 ± 0.12 bb	0.95 ± 0.09	1.16 ± 0.13	0.95 ± 0.06

ALT, alanine aminotransferase; AST, aspartate aminotransferase; Chol, cholesterol; HDLc, high-density lipoprotein cholesterol; IL, interleukin; LDLc, low-density lipoprotein cholesterol; oxLDL, oxidized LDL; TG, triglycerides. Results are presented as mean ± SEM. a *p* < 0.05 vs. Ctrl group; aa *p* < 0.01 vs. Ctrl group; aaa *p* ≤ 0.001 vs. Ctrl group; b *p* < 0.05 vs. Gd group; bb *p* < 0.01 vs. Gd group; bbb *p* ≤ 0.001 vs. Gd group for each time-point exposure.

## Data Availability

The original contributions presented in this study are included in the article/Supplementary Materials. The raw data supporting the conclusions of this article will be made available by the authors upon request. Further inquiries can be directed at the corresponding authors.

## Supplementary Materials

The following supporting information can be downloaded at: https://www.mdpi.com/article/10.3390/jox15020034/s1, Table S1: List of all the differentially expressed genes between the control, Gd (III) and DOTA groups after the short-term exposure to the compounds; Table S2: List of all the differentially expressed genes between the control, Gd (III) and DOTA groups after the long-term exposure to the compounds; Table S3: List of the significant Gene Ontology terms obtained by the enrichment analysis of all differentially expressed genes after the short-term exposure to the compounds; Table S4: List of the significant Gene Ontology terms obtained by the enrichment analysis of all differentially expressed genes after the long-term exposure to the compounds.; Table S5: List of the significant Kyoto Encyclopedia of Genes and Genomes terms obtained by the enrichment analysis of all differentially expressed genes after the short-term exposure to the compounds; Table S6: List of the significant Kyoto Encyclopedia of Genes and Genomes terms obtained by the enrichment analysis of all differentially expressed genes after the long-term exposure to the compounds.

## Abbreviations

The following abbreviations are used in this manuscript:

GBCA	gadolinium-based contrast agents
MRI	magnetic resonance imaging
Gd (III)	gadolinium
CKD	chronic kidney disease
NSF	nephrogenic systemic fibrosis
DOTA	gadoteric acid
DGE	differential gene expression
AST	aspartate aminotransferase
ALT	alanine aminotransferase
TG	triglycerides
HDLc	high-density lipoprotein cholesterol
LDLc	low-density lipoprotein cholesterol
oxLDL	oxidized LDL
IL6	interleukin 6
ELISA	enzyme-linked immunosorbent assays
GO-BP	gene ontology–biological process
KEGG	kyoto encyclopedia of genes and genomes
PLT	platelets
MPV	mean platelet volume
RBC	red blood cells
MCHC	mean corpuscular hemoglobin concentration
M-DCs	myeloid-derived dendritic cells
MMP	metalloproteinase
